# Characterization of the metabolism of eupalinolide A and B by carboxylesterase and cytochrome P450 in human liver microsomes

**DOI:** 10.3389/fphar.2023.1093696

**Published:** 2023-01-25

**Authors:** Yingzi Li, Xiaoyan Liu, Ludi Li, Tao Zhang, Yadong Gao, Kewu Zeng, Qi Wang

**Affiliations:** ^1^ Department of Toxicology, School of Public Health, Peking University, Beijing, China; ^2^ State Key Laboratory of Natural and Biomimetic Drugs, School of Pharmaceutical Sciences, Peking University, Beijing, China; ^3^ Key Laboratory of State Administration of Traditional Chinese Medicine for Compatibility Toxicology, Beijing, China; ^4^ Key Laboratory of Toxicological Research and Risk Assessment for Food Safety, Beijing, China

**Keywords:** eupalinolide A, eupalinolide B, human liver microsomes, carboxylesterase, cytochrome P450, metabolic stability, enzyme kinetics, CYP phenotyping

## Abstract

Eupalinolide A (EA; Z-configuration) and eupalinolide B (EB; E-configuration) are bioactive cis-trans isomers isolated from *Eupatorii Lindleyani Herba* that exert anti-inflammatory and antitumor effects. Although one pharmacokinetic study found that the metabolic parameters of the isomers were different in rats, metabolic processes relevant to EA and EB remain largely unknown. Our preliminary findings revealed that EA and EB are rapidly hydrolyzed by carboxylesterase. Here, we investigated the metabolic stability and enzyme kinetics of carboxylesterase-mediated hydrolysis and cytochrome P450 (CYP)-mediated oxidation of EA and EB in human liver microsomes (HLMs). We also explored differences in the hydrolytic stability of EA and EB in human liver microsomes and rat liver microsomes (RLMs). Moreover, cytochrome P450 reaction phenotyping of the isomers was performed *via in silico* methods (i.e., using a quantitative structure-activity relationship model and molecular docking) and confirmed using human recombinant enzymes. The total normalized rate approach was considered to assess the relative contributions of five major cytochrome P450s to EA and EB metabolism. We found that EA and EB were eliminated rapidly, mainly by carboxylesterase-mediated hydrolysis, as compared with cytochrome P450-mediated oxidation. An inter-species difference was observed as well, with faster rates of EA and EB hydrolysis in rat liver microsomes. Furthermore, our findings confirmed EA and EB were metabolized by multiple cytochrome P450s, among which CYP3A4 played a particularly important role.

## 1 Introduction

**FIGURE 1:**
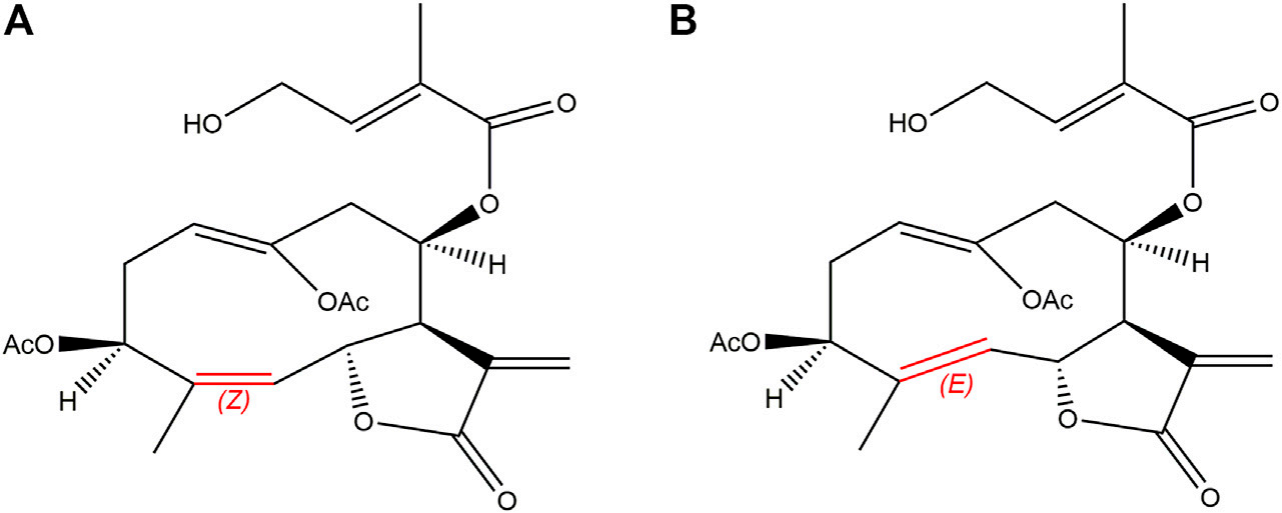
Chemical structures of EA [**(A)**; Z-configuration] and EB [**(B)**; E-configuration].

Eupalinolide A (EA) and eupalinolide B (EB), a pair of cis-trans isomers (Figure 1), are bioactive sesquiterpenoids found in *Eupatorii Lindleyani Herba* (Wang X. et al., 2020). As potential drug candidates, these isomers have been reported to exert anti-inflammatory and antitumor effects (Zhang et al., 2022a; Zhang et al., 2022b; Yang et al., 2022). Recently, EB was reported to exert anti-neuroinflammatory activity *via* targeting ubiquitin-specific protease 7 in the setting of neurodegenerative disease (Zhang et al., 2022c).

Currently, the United States Food and Drug Administration (FDA) recommended that all new chemical entities under development be identified on the basis of their metabolic properties before administration to humans^1^. Assessment of candidate compound pharmacokinetics and pharmacodynamics is critical for successful drug development (Kumar and Surapaneni, 2001; Meng and Liu, 2014). Poor metabolic stability *in vitro* is generally predictive for unfavorable pharmacokinetic properties such as rapid compound metabolism *in vivo*, low bioavailability and short duration of action (Liang et al., 2015). To date, few studies have investigated the metabolism of EA and EB. One pharmacokinetic study in rats revealed that these isomers exhibit different metabolic parameters, with EB having a higher bioavailability and thus greater potential for clinical use (Zhang et al., 2015). Importantly, significant differences exist between human and rodent enzymatic function and metabolism. As an ideal *in vitro* human-based test system, human liver microsomes (HLMs) offer numerous advantages for evaluating drug metabolism such as low cost, robustness, low lot-to-lot variability and wide commercial availability (Harper and Brassil, 2008; Miners et al., 2010; Caldwell and Yan, 2014). Our preliminary findings revealed that EA and EB are largely metabolized by carboxylesterase. In this study, we investigated the metabolic stability and kinetics of EA and EB in HLMs, focusing particularly on cytochrome P450 (CYP)-mediated oxidation and carboxylesterase-mediated hydrolysis. We also compared the hydrolytic stability of EA and EB in HLMs and rat liver microsomes (RLMs) to explore possible inter-species differences in metabolism among humans and rats.

Enzyme phenotyping, another important step in the drug development process, is performed to avoid undesirable drug interactions. Recombinant human cytochrome P450s (rhCYPs) are widely used for enzyme phenotyping in early drug development (Chen et al., 2011). In addition, advances in computing methods such as quantitative structure-activity relationship (QSAR) model construction and molecular docking analysis have facilitated isozyme prediction. A QSAR model is established based on structural characteristics and properties of known compounds and is used to predict metabolism of compounds with similar structures under the assumption that molecules with similar structures likely exhibit similar biochemical properties (Wu et al., 2013). The FDA as well as the Registration, Evaluation and Authorization of Chemicals issued by the European Union consider QSAR predictions to be highly reliable (Gertrudes et al., 2012; Hong et al., 2016). Molecular docking, another computational approach, has become widely used in the early stage of drug development to simulate interactions among proteins and ligands for the purposes of elucidating compound enzymatic metabolism (Kaur et al., 2019; Kazmi et al., 2019).

In this study, we investigated the metabolic stability and kinetics of CYP-mediated oxidation and carboxylesterase-mediated hydrolysis of EA and EB using HLMs. Moreover, CYP phenotyping of the isomers was explored using *in silico* methods including a QSAR model and molecular docking analysis. Findings were confirmed with rhCYPs.

## 2 Materials and methods

### 2.1 Reagents and materials

EA (purity ≥ 98%) and sulfaphenazole were purchased from Beijing Mreda Technology Co., Ltd. (Beijing, China), while EB (purity ≥ 98%) was purchased from Chengdu Push Biotechnology Co., Ltd. (Chengdu, China). Carbamazepine was purchased from Shanghai Yuanye Biotechnology Co., Ltd. (Shanghai, China). Nicotinamide adenine dinucleotide phosphate (NADPH) was purchased from Sigma Aldrich (St. Louis, MO, United States). Midazolam was purchased from Beijing Gersion Biotechnology Co., Ltd. (Beijing, China). Phenacetin and tolbutamide were purchased from Aladdin Industrial Corp. (Shanghai, China). (S)-Mephenytoin, dextromethorphan, dextrorphan and 1′-OH-midazolam were purchased from Glpbio (Montclair, CA, United States). Paracetamol and bis(4-nitrophenyl) phosphoricacid (BNPP) were purchased from Beijing Innochem Technology Co., Ltd. (Beijing, China). The 4-OH-tolbutamide was purchased from J&K Scientific Technology Co., Ltd. (Beijing, China), while 4-OH-mephenytion was purchased from Bioplastics (Landgraaf, Netherlands). Acetonitrile and methanol for high-performance liquid chromatography (HPLC) and mass spectrometry (MS) were obtained from Thermo Fisher Scientific (Waltham, MA, United States). All other chemicals and reagents, including MgCl_2_, potassium phosphate dibasic and dimethyl sulfoxide, were of analytical grade and commercially available.

Pooled HLMs were purchased from Xeno Tech (Kansas City, KS, United States); HLM donors were individuals without hepatic disease and included 100 men and 100 women aged 16–78 years (lot no. 1910096). Pooled RLMs were purchased from IPHASE (Beijing, China). Activity of HLM and RLM was confirmed *via* the detection of major metabolic enzymes. The cDNA-expressed human CYP1A2, 2C9, 2C19, 2D6 and 3A4 were purchased from Cypex (Dundee, United Kingdom).

### 2.2 Metabolic stability of EA and EB oxidation and hydrolysis in HLMs

#### 2.2.1 Determination of flavin-containing monooxygenase (FMO) contribution to EA and EB metabolism

To explore CYP-mediated oxidation, we first determined how FMO affects EA and EB metabolism. An incubation system consisting of 100 mM phosphate buffer solution at pH 7.4, HLMs (0.5 g/L), MgCl_2_ (5 mM) and NADPH (1 mM) was utilized. The mixture was preincubated at 37°C for 5 min. To initiate the metabolic reaction, EA or EB (dissolved in methanol at a final concentration of 10 μM) was added. To inactivate FMO, HLMs were heated at 50°C for 90 s (Zhuang et al., 2014). HLMs in positive control group were not subjected to heat treatment while negative control groups contained deactivated HLMs. Organic solvent content in the incubation system did not exceed 0.5%. The metabolic reaction was terminated by adding 200 µl of stop solution [ice-cold acetonitrile/methanol (1:1) containing internal standard (IS) carbamazepine] at 0, 2, 5, 10, 20, and 30 min, respectively. Samples were vortexed and centrifuged at 17,000 g for 15 min. Supernatant was collected and subjected to HPLC analysis. All incubations were performed in triplicate.

Data were expressed as means ± SD and analyzed using SPSS software (IBM, New York, NY, United States). Student’s t*-*test was used to compare differences between groups at various time points, and *p* < 0.05 was considered to be statistically significant.

#### 2.2.2 Selection of carboxylesterase inhibitor BNPP concentration in HLMs

To determine ideal BNPP concentration for inhibiting carboxylesterase activity in HLMs, an incubation system consisting of 100 mM phosphate buffer solution at pH 7.4, HLMs (0.5 g/L), MgCl_2_ (5 mM) and BNPP (0.5, 1, 1.5 and 2 mM) was prepared (Zhuang et al., 2014; Wang Y. Q. et al., 2020; Zhang et al., 2022d; Jin et al., 2022). The mixture was preincubated at 37°C for 5 min and the metabolic reaction was initiated by addition of EA or EB (at a final concentration of 10 μM). Positive control groups lacked BNPP while negative control groups contained deactivated HLMs. Organic solvent content in the incubation system did not exceed 0.5%. The above reaction was terminated by adding 200 µl of ice-cold stop solution at 30 min. Sample preparation and evaluation were as described in 2.2.1. All incubations were performed in triplicate. The percentage of EA or EB hydrolysis inhibition was calculated using the following Eq. 1:

$$Inhibition\%=\left[1-\left(∆substrate\;in\;the\;presence\;of\;chemical\;inhibitor/∆substrate\;in\;the\;absence\;of\;chemical\;inhibitor\right)\right]\times 100\%$$ (1)

Data were expressed as means ± SD and analyzed using SPSS. Student’s *t-*test was used to compare differences between groups, and *p* < 0.05 was considered to be statistically significant.

#### 2.2.3 Metabolic stability of EA and EB in HLMs to oxidation and hydrolysis

The metabolic stability of EA and EB was determined by using the most conventional method of measuring test compound depletion over time (Caldwell and Yan, 2014). For determination of metabolic stability to oxidation, a system composed of 100 mM phosphate buffer solution at pH 7.4, HLMs (0.5 g/L), MgCl_2_ (5 mM), NADPH (1 mM) and BNPP (0.5 mM) was preincubated at 37°C for 5 min. Then, EA or EB (at a final concentration of 10 μM) was added to initiate the metabolic reaction. For determination of metabolic stability to hydrolysis, the incubation system was prepared either without NADPH or BNPP (mixture containing NADPH and lacking BNPP evaluated CYP and carboxylesterase co-mediated metabolic reactivity). Organic solvent content in the incubation system did not exceed 0.5%. Negative control groups contained deactivated HLMs. The above reactions were terminated by adding 200 µl of ice-cold stop solution at 0, 2, 5, 10, 20, and 30 min, respectively. Sample preparation and determination were as described in 2.2.1. All incubations were performed in triplicate.

The natural logarithm of the remaining percentage of EA or EB and reaction time were plotted to obtain the slope (*k*) by linear regression; EA and EB elimination half-lives (*t*_1/2_) were calculated using Eq. 2. The well-stirred model (Obach, 1997; Slaughter et al., 2003; Reddy et al., 2005; Zhao et al., 2005) was used to extrapolate both intrinsic (*CL*_int_) [ml/(min·kg)] and hepatic (*CL*_h_) [ml/(min·kg)] clearance of EA and EB from the human liver.

$$t_{1/2}=-0.693/k$$ (2)

$$CL_{\mathrm{int}}=0.693/t_{1/2}\left(G_{HLM}/W_{liver}\right)\left(W_{liver}/W_{body}\right)/C_{protein}$$ (3)

$$CL_{h}=\frac{Q_{h}\times {CL}_{\mathrm{int}}}{Q_{h}+{CL}_{\mathrm{int}}}$$ (4)

G_HLM_ represents average liver microsomal protein concentration (mg); W_liver_ represents liver weight (g); W_body_ represents body weight (kg); C_protein_ represents reaction system protein concentration (mg/mL); *Q*_h_ represents liver blood flow velocity. Empirical values of relevant human physical and chemical parameters were 48.8, 25.7 and 20.7 for W_liver_/W_body_, G_HLM_/W_liver_ and *Q*_h_, respectively (Slaughter et al., 2003).

### 2.3 Kinetics of EA and EB oxidation and hydrolysis in HLMs

Experiments were conducted under conditions of linear substrate depletion (0.5 g/L HLMs; 10 min) (Zhang et al., 2008). For assay of CYP-mediated oxidation kinetics, HLMs (0.5 g/L) were incubated with NADPH (1 mM), BNPP (0.5 mM), MgCl_2_ (5 mM) and 100 mM phosphate buffer solution at pH 7.4. After preincubation at 37°C for 5 min, the metabolic reaction was initiated by adding EA or EB (2.5, 5, 10, 25, 50 or 100 µM). For assay of carboxylesterase-mediated hydrolysis kinetics, the incubation system was prepared lacking either NADPH or BNPP. Negative controls contained deactivated HLMs. Organic solvent content in the incubation system did not exceed 0.5%. The above reactions were terminated by adding 200 µl of ice-cold stop solution at 10 min. Sample preparation and evaluation were as described in 2.2.1. All incubations were performed in triplicate.

The elimination rate of EA and EB was fit with Michaelis-Menten kinetics. Enzyme kinetic parameters, the Michaelis–Menten constant (*K*_m_) and maximum velocity (*V*_max_) were calculated using GraphPad Prism 7 to obtain a non-linear least-square fit to the Michaelis-Menten equation. *In vitro CL*_int_ was calculated as *CL*_int_ = *V*_max_/*K*_m_ (Yu et al., 2013).

### 2.4 Hydrolytic stability of EA and EB in RLMs

Male and female RLMs were mixed in a 1:1 ratio for incubation. A system composed of 100 mM phosphate buffer solution at pH 7.4, RLMs (0.5 g/L), MgCl_2_ (5 mM) and NADPH (1 mM) was preincubated at 37°C for 5 min. Then, EA or EB (at a final concentration of 10 μM) was added to initiate hydrolysis. Procedures were as described in 2.2.3. All incubations were performed in triplicate. Hydrolytic stability parameters were calculated as described in 2.2.3. Empirical values of relevant rat physical and chemical parameters were 44.8, 40 and 55.2 for W_liver_/W_body_, G_HLM_/W_liver_ and *Q*_h_, respectively (Slaughter et al., 2003).

### 2.5 CYP phenotyping

#### 2.5.1 QSAR model prediction of CYP phenotyping

The QSAR-based software ADMET Predictor 8.5 (Simulation Plus, Lancaster, CA, United States) was used to predict metabolic phenotyping for CYP1A2, CYP2A6, CYP2B6, CYP2C8, CYP2C9, CYP2C19, CYP2D6, CYP2E1 and CYP3A4 as relevant to EA and EB. The 2D structures of EA and EB were input into the software in MDL Mol file format and analyzed in metabolic modules.

#### 2.5.2 Molecular docking analysis of CYP isozymes

The molecular docking software SYBYL-X 2.0 (Tripos, St Louis, MO, United States) was used to determine whether EA and EB could bind each of the five main CYP isoforms (CYP1A2, CYP2C9, CYP2C19, CYP2D6 and CYP3A4). The 3D structures of proteins [PDB ID: 2HI4 (CYP1A2), 5W0C (CYP2C9), 4GQS (CYP2C19), 3TBG (CYP2D6), and 6MA7 (CYP3A4)] were retrieved from the Protein Data Bank. Protein pretreatment involved removal of metal ions, removal of water and solvent molecules, addition of hydrogen atoms, as well as repair of side-chains and side-chain amides. The 3D structures of EA and EB, which were used as ligands, were input into SYBYL-X in mol2 format. A Gasteger-Hückel charge was added to the third-order force field to minimize EA and EB energy. Surflex-Dock Geom mode was used for molecular docking. The root-mean-square deviation (RMSD) is the average distance between the highest-ranking docked and reference structures. A protein is considered appropriate for molecular docking when the RMSD value is less than 2 Å. The total score is the most important evaluation index for molecular docking, which comprehensively considers polar complementarity, solvation terms, entropic terms and hydrophobic complementarity. It is considered a stable interaction when the total score is greater than 6 (Gao et al., 2016).

#### 2.5.3 Determination of rhCYP activity

Each of the probe substrates (50 µM phenacetin for CYP1A2; 120 µM tolbutamide for CYP2C9; 40 µM (S)-mephenytoin for CYP2C19; 5 µM dextromethorphan for CYP2D6; 5 µM midazolam for CYP3A4) was incubated with 100 pmol ml^−1^ of rhCYPs (CYP1A2, 2C9, 2C19, 2D6 and 3A4) for 30 min. The incubation system also included MgCl_2_ (5 mM) and 100 mM potassium phosphate buffer at pH 7.4. Control groups were treated with stop solution prior to rhCYP addition. Organic solvent content in the incubation system did not exceed 0.5%. Sample preparation was as described in 2.2.1. Supernatant was collected and analyzed for specific metabolites *via* liquid chromatography-tandem mass spectrometry (LC-MS/MS). All incubations were performed in triplicate.

#### 2.5.4 CYP phenotyping of EA and EB with rhCYPs

Because CYP1A2, 2C9, 2C19, 2D6 and 3A4 are the most important human CYP isoforms involved in drug metabolism (Zhang et al., 2008), we investigated whether the above isoforms were the metabolic enzymes of these isomers. Either EA or EB (10 μM) was incubated with each of the rhCYPs (100 pmol ml^−1^; CYP1A2, 2C9, 2C19, 2D6 or 3A4), NADPH (1 mM) and MgCl_2_ (5 mM) in 100 mM potassium phosphate buffer at pH 7.4 for 10 min. Control groups contained deactivated rhCYPs. Organic solvent content in the incubation system did not exceed 0.5%. Sample preparation and determination were as described in 2.2.1. All incubations were performed in triplicate.

Relative contribution of CYP isoforms to EA and EB metabolism was estimated using the total normalized rate (TNR) approach as described by Rodrigues (Rodrigues, 1999) and as shown in Eq. 5. The metabolism rate (pmol/min/pmol CYP) for each rhCYP isoform (rCYP_n_) was multiplied by the mean specific content of the corresponding CYP isoform in native HLMs (mCYP_n_) to yield the normalized rate (NR). Then, NR values were summed to obtain the TNR and percentages of TNRs calculated for each rCYP_n_.

$$TNR\%=\frac{NR}{TNR}\times 100=\frac{pmol/\min/pmolr{CYP}_{n}\times pmolm{CYP}_{n}/mg}{\sum \left(pmol/\min/pmolr{CYP}_{n}\times pmolm{CYP}_{n}/mg\right)}\times 100$$ (5)

### 2.6 Determination of residual EA and EB by HPLC

Quantification of EA and EB was accomplished *via* HPLC using an Agilent 1200 Infinity series instrument (Waldbronn, Germany) fitted with a Zorbax SB-C18 HPLC column (4.6 mm × 150 mm, 5 μm; Agilent, Santa Clara, CA, United States). Column temperature was maintained at 40°C with a mobile phase flow rate of 1 ml/min. The detection wavelength was 220 nm. The two compounds were separated by isocratic elution with a mobile phase consisting of 30% acetonitrile and 70% H_2_O. Retention times of EA and EB were 13.55 min and 20.91 min, respectively. Incubation sample EA and EB concentrations were quantified using standard curves prepared from samples over a concentration range of 0.5–100 µM.

### 2.7 Quantification of CYP isoform activity by UFLC‒MS/MS

The quantification of five CYP-specific substrate metabolites (paracetamol for CYP1A2; 4-OH-tolbutamide for CYP2C9; 4-OH-mephenytion for CYP2C19; dextrorphan for CYP2D6; 1′-OH-midazolam for CYP3A4) was performed according to a method previously reported but with slight modification (Shen et al., 2013). Metabolites were analyzed by a UFLC‒MS/MS 8050 system (Shimadzu Corp., Kyoto, Japan) consisting of an LC-30AD binary pump, an SPD M30A PDA detector, an SIL-30AC autosampler, a CTO-20AC column oven and an 8,050 triple quadrupole mass spectrometer outfitted with a heated ESI source. Samples were separated on an ACQUITY UPLC^®^ BEH Shield RP-C_18_ VanGuard™ column (100 mm × 2.1 mm, 1.7 μm; Waters, Milford, MA, United States) with an ACQUITY UPLC^®^ BEH Shield RP-C18 VanGuard™ precolumn (5 mm × 2.1 mm, 1.7 μm; Waters, Milford, MA, United States).

## 3 Results

### 3.1 HPLC method validation

**FIGURE 2:**
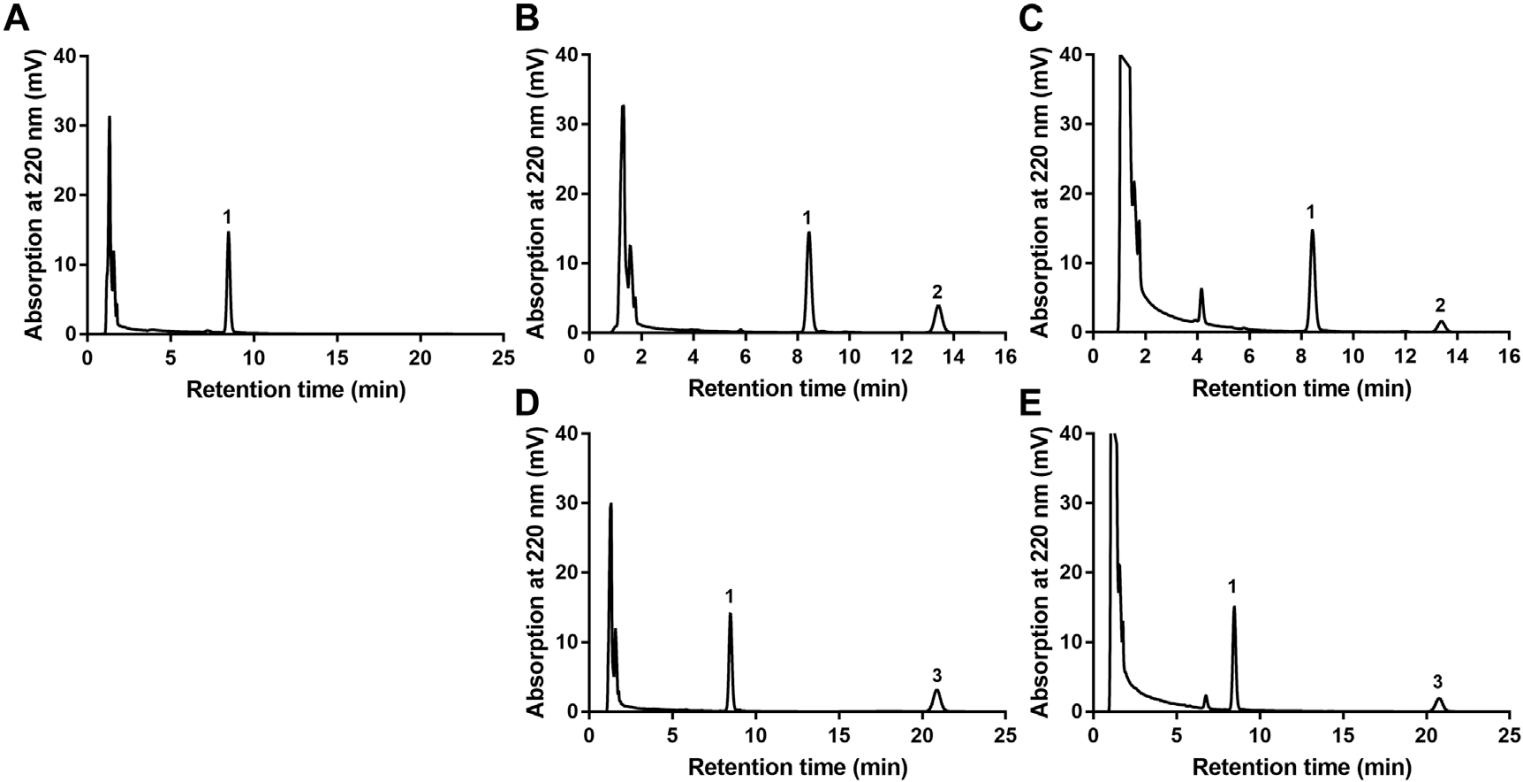
HPLC chromatograms of EA or EB in HLMs. **(A)** blank HLMs; **(B)** blank HLMs spiked with EA; **(C)** incubation of EA with HLMs; **(D)** blank HLMs spiked with EB; **(E)** incubation of EB with HLMs; 1: IS, 2: EA, 3: EB.

The newly developed detection method of EA or EB in HLMs was robust. As shown in Figure 2, endogenous substances within HLMs did not interfere with EA, EB or IS quantification, which were completely separated with good peak shape. Calibration curve regression equations were Y = 24.823X + 0.5169, r^2^ = 0.9999 for EA, and Y = 18.215X + 0.73, r^2^ = 0.9998 for EB over a range of 0.5–100 µM. The limit of quantification (LOQ) of the method used was 0.5 µM. Intraday and interday precision were within 0.62% and 6.84% for EA, and 1.16% and 5.86% for EB, respectively, at low, middle and high levels of quality control (1.5, 10 and 75 μM; *n* = 3). Accuracy ranged from −1.23%−8.77% for EA and 2.86%–9.51% for EB. The extraction recoveries of EA and EB were over a range of 95.89%–104.13% and 95.09%–106.04%, respectively, at the above quality control concentrations. The matrix effect was within the range of 95.54%–102.51% for EA and 102.28%–106.74% for EB. The two isomers were stable at ambient temperature for 6 h, at −20°C for 3 days or after three freeze-thaw cycles.

### 3.2 FMO was not involved in EA and EB metabolism

**FIGURE 3:**
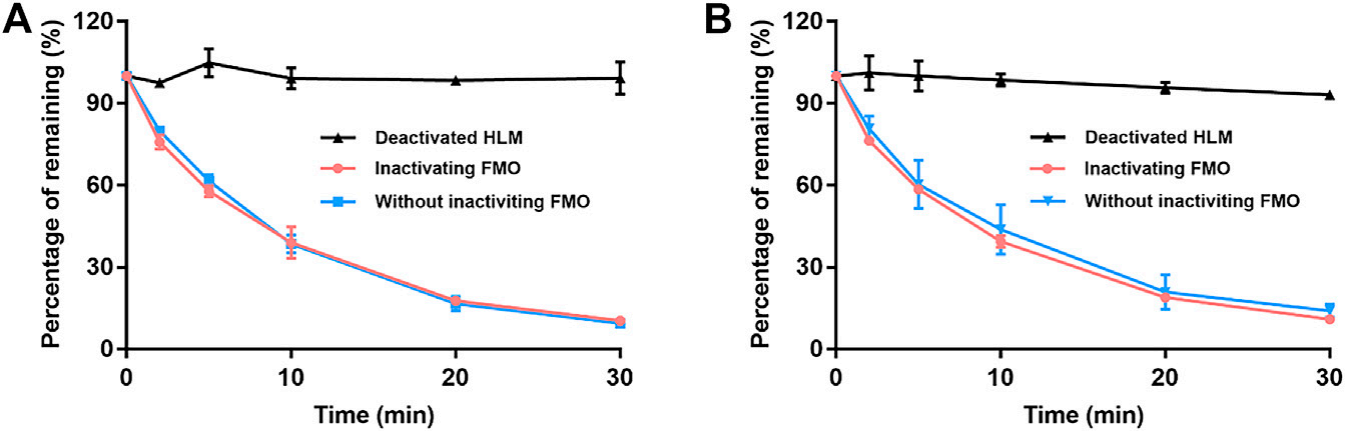
The contribution of FMO to EA **(A)** and EB **(B)** metabolism in HLMs with NADPH. Deactivated HLMs were used as negative control (mean ± SD; *n* = 3).

Figure 3 details the depletion profiles of EA and EB in HLMs with or without FMO inactivation. No significant differences in EA or EB elimination at each time point were noted whether or not FMO was inactivated by heat treatment. Our findings suggest that FMO was not involved in EA or EB metabolism. These results were consistent with the fact that the molecular structures of the two isomers do not contain heteroatoms such as nucleophilic nitrogen, sulfur or phosphorus atoms, which can be oxidized by FMO. Thus, FMO was not inactivated in subsequent experiments.

### 3.3 BNPP concentration selection in HLMs

**FIGURE 4:**
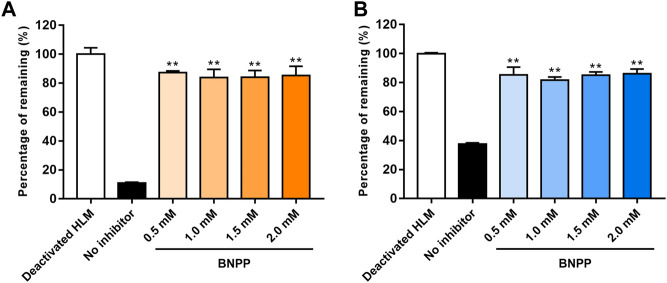
Influence of BNPP on EA **(A)** and EB **(B)** stability in HLMs in the absence of NADPH. Incubation with deactivated HLMs was performed for negative controls (100% remained). Incubation of HLMs without inhibition was performed for positive control. Significant differences from positive control were analyzed using the *t*-test. *p < 0.05, **p < 0.01 (mean ± SD; *n* = 3).

The influence of different BNPP concentrations on EA and EB stability in the absence of NADPH is shown in Figure 4. Compared with the positive control group (without inhibitor), hydrolysis elimination rates of EA and EB decreased significantly in the presence of BNPP. The strongest inhibitory effect on hydrolysis of EA was observed at a BNPP concentration of 0.5 mM (inhibitory percentage of 87.38%). The greatest inhibitory rate of EB hydrolysis was observed at a BNPP concentration of 2 mM (86.11%). Our findings suggest that carboxylesterase was the main enzyme responsible for hydrolysis of EA and EB in HLMs. Moreover, 0.5 mM BNPP was selected to inhibit carboxylesterase activity and thus EA and EB hydrolysis in HLMs.

### 3.4 Stability of EA and EB to oxidation and hydrolysis in HLMs

**FIGURE 5:**
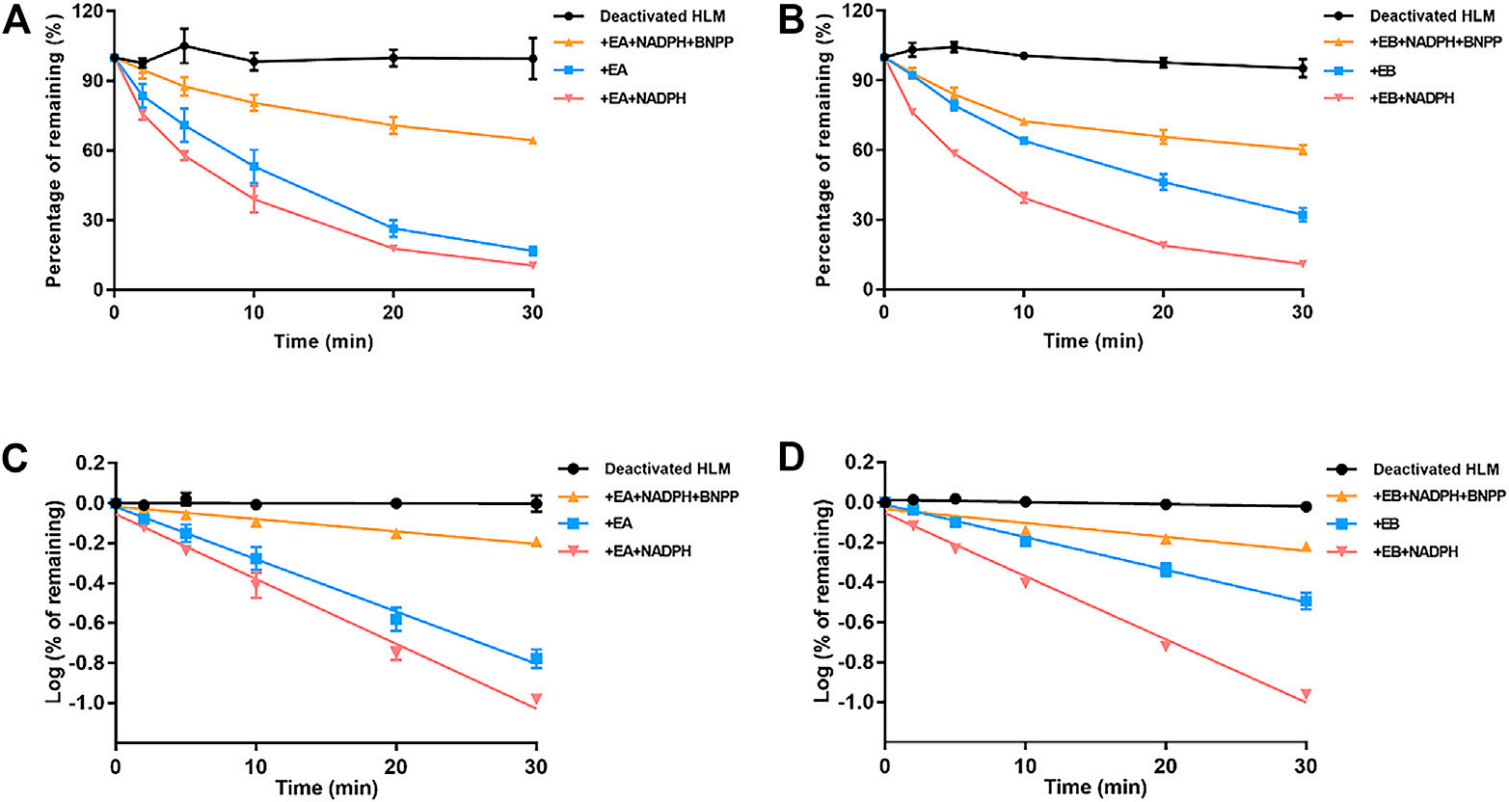
Contributions of carboxylesterase and CYP to EA **(A,C)** and EB **(B,D)** metabolism in HLMs. Deactivated HLMs were used as negative control (mean ± SD; *n* = 3).

Stability of EA and EB to oxidation and hydrolysis in HLMs was evaluated at a concentration of 10 μM by incubation with and without NADPH or BNPP. Plots of percentages of EA and EB remaining in HLMs versus time are shown in Figure 5. In control samples with deactivated HLMs, negligible reductions in EA and EB were noted, suggesting that the non-specific protein binding can be ignored. Moreover, addition of BNPP to the incubation system markedly slowed EA and EB depletion, indicating that HLM metabolism of EA and EB occurred mainly by hydrolysis. The metabolism rate and clearance parameters of the isomers are shown in Table 1. In the presence of BNPP, the *t*_1/2_ of EA increased from 26.70 ± 2.93 to 111.36 ± 5.55 min, and of EB from 42.66 ± 3.09 to 94.50 ± 6.36 min, suggesting that rapid metabolism of EA and EB occurred primarily by carboxylesterase. Furthermore, our findings confirmed metabolic stereoselectivity of EA and EB.

**TABLE 1:** Metabolic clearance parameters of EA and EB incubated with HLMs in the absence or presence of NADPH, or inhibitor of carboxylesterase BNPP (mean ± SD; *n* = 3).

Group	Enzyme involved	*t*_1/2_ (min)	*CL*_int_ [ml/(min·kg)]	*CL*_h_ [ml/(min·kg)]	Metabolism rate (%)
+EA+NADPH+BNPP	CYP	111.36 ± 5.55	8.90 ± 0.77	9.04 ± 0.25	55.32 ± 1.20
+EA	Carboxylesterase	26.70 ± 2.93	65.63 ± 7.15	15.71 ± 0.41	83.26 ± 1.80
+EA+NADPH	CYP and carboxylesterase	21.37 ± 0.40	81.35 ± 1.53	16.50 ± 0.06	89.56 ± 0.60
+EB+NADPH+BNPP	CYP	94.50 ± 6.36	18.44 ± 1.24	9.75 ± 0.35	47.98 ± 4.00
+EB	Carboxylesterase	42.66 ± 3.09	40.89 ± 2.85	13.73 ± 0.33	67.82 ± 3.00
+EB+NADPH	CYP and carboxylesterase	21.93 ± 0.30	79.26 ± 1.09	16.41 ± 0.05	89.07 ± 0.40

### 3.5 Kinetics of EA and EB oxidation and hydrolysis in HLMs

**FIGURE 6:**
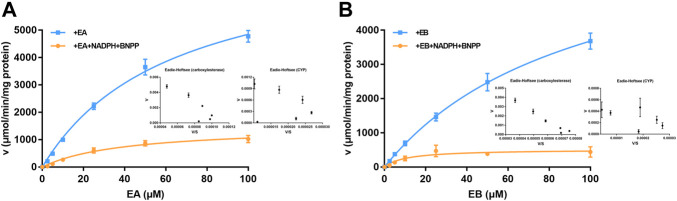
Michaelis–Menten curves of EA **(A)** and EB **(B)** in HLMs metabolized by carboxylesterase or CYP and their corresponding Eadie-Hofstee plots (as insert) (mean ± SD; *n* = 3).

Michaelis–Menten curves of EA and EB HLM metabolism by carboxylesterase or CYP are shown in Figure 6; parameters relevant to oxidation and hydrolysis of EA and EB in HLMs are listed in Table 2. The values of carboxylesterase-mediated *CL*_int_ of EA and EB were 124.75 ± 9.56 and 75.00 ± 11.99 L/(min·mg), respectively, which were markedly higher than corresponding clearance values of CYP (36.87 ± 3.12 and 49.26 ± 15.47 L/(min·mg) for EA and EB). The above results were consistent with metabolic stability findings and suggest that hydrolysis by carboxylesterase played a dominant role in EA and EB metabolism. Moreover, differences in *K*_m_ and *V*_max_ between EA and EB further highlight metabolic stereoselectivity of the isomers.

**TABLE 2:** Oxidative and hydrolysis kinetic parameters of EA and EB in HLMs (mean ± SD; *n* = 3).

	Compound	*K*_m_ (µM)	*V*_max_ (µmol/min/mg protein)	*CL*_int_ [L/(min·mg)]
Oxidative kinetic parameters	EA	41.02 ± 8.05	1,497.67 ± 186.98	36.87 ± 3.12
	EB	10.68 ± 1.02	515.97 ± 124.03	49.26 ± 15.47
hydrolysis kinetic parameters	EA	63.86 ± 3.88	7,945.00 ± 291.66	124.75 ± 9.56
	EB	104.86 ± 37.46	7,567.00 ± 1471.38	75.00 ± 11.99

### 3.6 Hydrolytic stability of EA and EB in HLMs and RLMs

**FIGURE 7:**
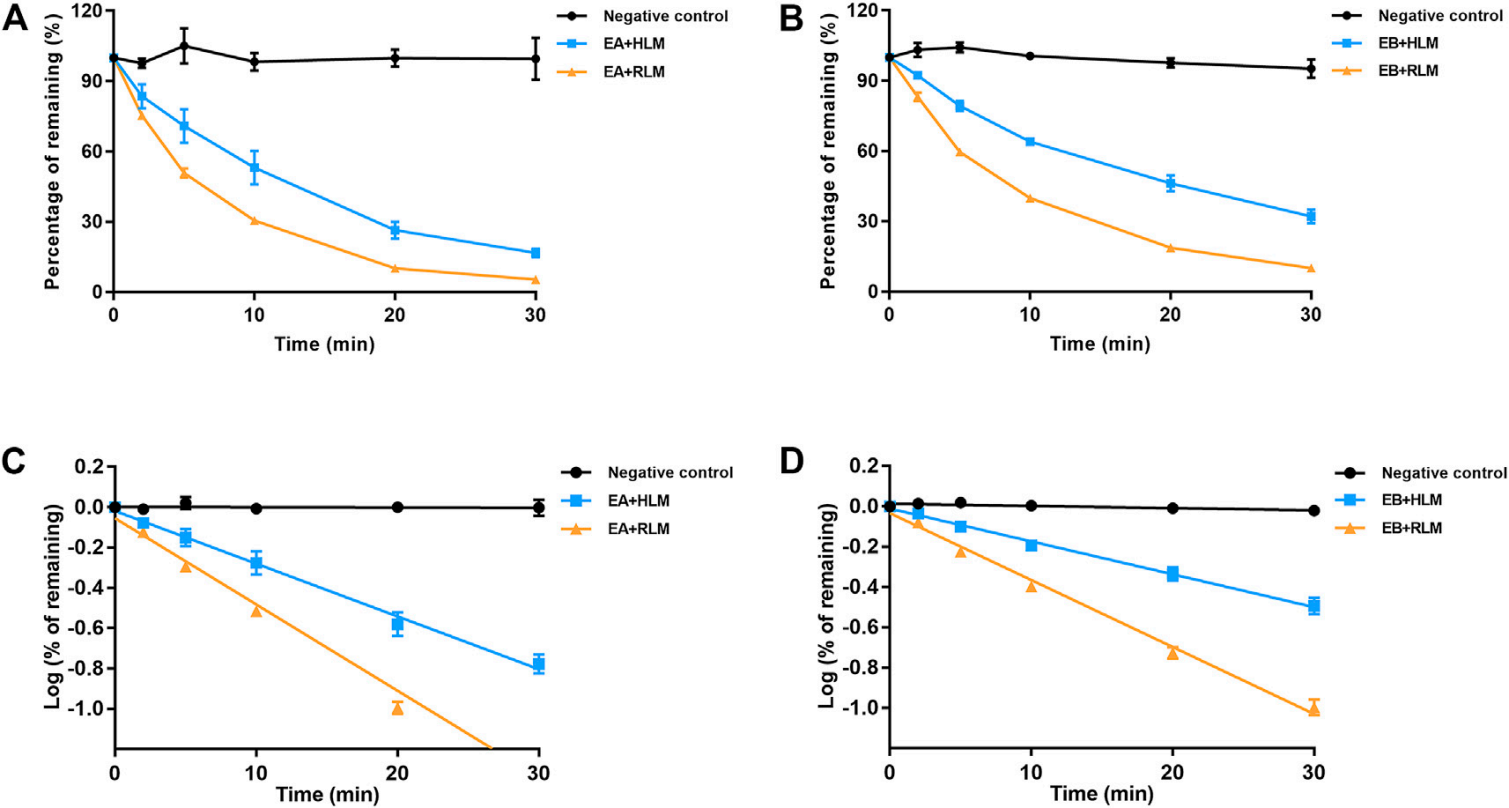
Comparison of EA **(A,C)** and EB **(B,D)** hydrolysis in HLMs or RLMs. Deactivated microsomes were used as negative control (mean ± SD; *n* = 3).

Because EA and EB were metabolized mainly by hydrolysis, we compared their hydrolytic stability in both HLMs and RLMs. Both isomers were hydrolyzed much more rapidly in RLMs than in HLMs (Figure 7). The *CL*_int_ values for EA were 65.63 ± 7.15 and 153.51 ± 4.94 [ml/(min·kg)] in HLMs and RLMs, respectively (Tables 1, 3). The *CL*_int_ values for EB were 40.89 ± 2.85 and 118.99 ± 4.69 [ml/(min·kg)] in HLMs and RLMs, respectively. Interestingly, EA was hydrolyzed more rapidly than EB both in HLMs and RLMs.

**TABLE 3:** Hydrolytic clearance parameters of EA and EB incubated with RLMs (mean ± SD; *n* = 3).

Compound	*t*_1/2_ (min)	*CL*_int_ [ml/(min·kg)]	*CL*_h_ [ml/(min·kg)]	Metabolism rate (%)
EA	16.19 ± 0.51	153.51 ± 4.94	40.60 ± 0.34	94.61 ± 0.4
EB	20.89 ± 0.81	118.99 ± 4.69	37.70 ± 0.46	89.90 ± 0.9

### 3.7 CYP phenotyping

#### 3.7.1 QSAR model prediction of CYP phenotyping

ADMET Predictor 8.5 was used to predict CYPs relevant to EA and EB. The prediction results for both isomers were identical, suggesting that CYP3A4 was involved in metabolism of both EA and EB.

#### 3.7.2 Molecular docking analysis

**FIGURE 8:**
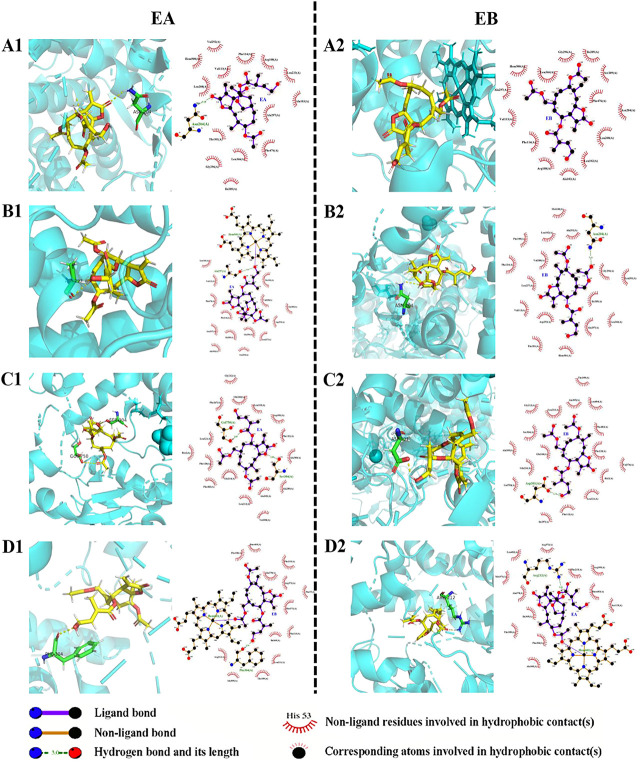
Interactions between EA (1) or EB (2) and CYP isoforms (left, 3D and right, 2D). **(A)** CYP2C9; **(B)** CYP2C19; **(C)** CYP2D6; **(D)** CYP3A4.

Molecular docking data for CYP2C9, CYP2C19, CYP2D6 and CYP3A4 are shown in Table 4 and Figure 8 (the RMSD of CYP1A2 did not meet docking standards and the result was excluded). These four enzymes had reasonable RMSD values less than 2 and total scores greater than 6, indicating that direct binding with EA and EB was likely. Bonds were formed primarily by hydrogen bonds; weak interactions were also involved. As such, CYP2C9, CYP2C19, CYP2D6 and CYP3A4 were likely involved in EA and EB metabolism.

**TABLE 4:** Interactions between EA or EB and CYP isozymes by molecular docking.

Compound	Isozymes	PDB ID	RMSD	Total score	H-bond number	Residues involved in H-bond formation
EA	CYP2C9	5W0C	1.73	10.33	1	A/Asn204
	CYP2C19	4GQS	1.03	11.89	1	A/Ala297
	CYP2D6	3TBG	1.49	8.38	2	A/Ser304、A/Gol750
	CYP3A4	6MA7	1.68	9.66	1	A/Arg212
EB	CYP2C9	5W0C	1.73	10.15	0	-
	CYP2C19	4GQS	1.03	9.37	1	A/Asn204
	CYP2D6	3TBG	1.49	7.99	1	A/Asp301
	CYP3A4	6MA7	1.68	10.57	1	A/Phe304

#### 3.7.3 CYP phenotyping of EA and EB with rhCYPs

**FIGURE 9:**
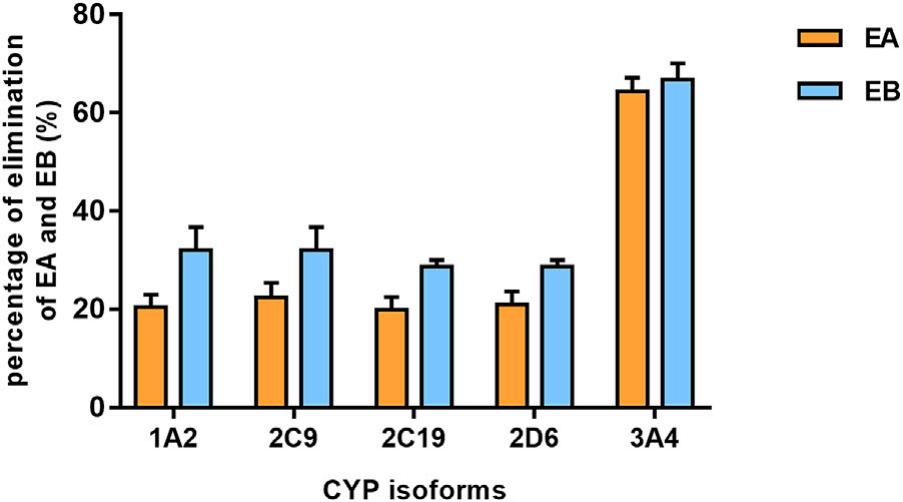
The elimination percentage of EA and EB after incubation with various cDNA-expressed human CYP isoforms for 30 min at 10 μmol L^−1^ (mean ± SD; *n* = 3).

Findings revealed that rhCYP isozyme activity was significant. As shown in Figure 9, both EA and EB were metabolized by human recombinant CYP1A2, CYP2C9, CYP2C19, CYP2D6 and CYP3A4, although to different extents. Table 5 shows the relative contributions of CYP isoforms involved in the metabolism of EA and EB. Findings for EA in descending order were as follows: CYP3A4 (66.20%) > CYP2C9 (19.87%) > CYP1A2 (8.48%) > CYP2C19 (3.50%) > CYP2D6 (1.94%). Findings for EB in descending order were as follows: CYP3A4 (58.18%) > CYP2C9 (23.65%) > CYP1A2 (11.62%) > CYP2C19 (4.39%) > CYP2D6 (2.15%). Our results indicate that EA and EB were metabolized by multiple CYP isoforms, of which CYP3A4 was the main isozyme responsible for oxidative metabolism.

**TABLE 5:** Contributions of rhCYPs to EA or EB metabolism assessed by the TNR approach (*n* = 3).

Compound	CYPs	Metabolic rate/pmol·min^−1^/(pmol rhCYP)	Mean CYP content/pmol·mg^−1^ (protein)	Normalized metabolic rate/pmol·mg^−1^·min^−1^ (protein)	TNR relative contribution (%)
EA	1A2	0.96	45	43.00	8.48
	2C9	1.05	96	100.74	19.87
	2C19	0.93	19	17.76	3.50
	2D6	0.98	10	9.82	1.94
	3A4	3.11	108	335.60	66.20
EB	1A2	1.46	45	65.71	11.62
	2C9	1.39	96	133.69	23.65
	2C19	1.31	19	24.81	4.39
	2D6	1.22	10	12.17	2.15
	3A4	3.05	108	328.89	58.18

## 4 Discussion

We explored the metabolic elimination of EA and EB by carboxylesterase and CYP in HLMs. The CYP-mediated oxidation of EA and EB was DADPH-dependent. During this reaction, one O atom in O_2_ is incorporated into the substrate, while another is reduced to H_2_O *via* a proton supplied by NADPH (McLean et al., 2015). However, carboxylesterase-mediated hydrolysis does not require NADPH (Zhuang et al., 2014). In the setting of combined metabolism by carboxylesterase and CYP, *CL*_int_ values for EA and EB were 81.35 ml/(min·kg) and 79.26 ml/(min·kg), respectively. Generally, a drug is considered to have a high clearance if its hepatic clearance exceeds 14 ml/(min·kg) (Liang et al., 2015). As such, our findings suggest that both EA and EB exhibit high clearance. The low metabolic stability of these isomers implies that they are likely to exert short-acting effects; structural modification may be required to enhance their bioavailability. Based on our unpublished findings, EA and EB metabolites are primarily products of carboxylesterase-mediated hydrolysis, where the main hydrolytic sites are the three ester bonds on branched chains. Thus, the preservation of active groups and structural modification involving side-chain ester bonds may be the most important.

Our findings revealed that carboxylesterase-mediated hydrolysis was the main pathway *via* which EA and EB were metabolized, as opposed to CYP oxidation. Metabolic stability analysis revealed that the *CL*_int_ of hydrolysis was about seven times that of oxidation for EA and approximately twice that of oxidation for EB. Moreover, enzyme kinetic studies further confirmed the dominant role of carboxylesterase in the metabolism of these isomers with significantly higher hydrolytic *V*_max_ values as compared to oxidation in HLMs both for EA and EB. Co-administration of carboxylesterase inhibitors with the isomers should thus likely be avoided. Comparison of the hydrolytic stability of EA and EB between HLMs and RLMs revealed significant differences in the rate of hydrolysis between humans and rats. Many studies have suggested metabolic variation of carboxylesterase-substrate compounds among different species. This phenomenon was likely observed due to interspecies differences of distribution, substrate preference and inhibitor response of carboxylesterase (Wang X. et al., 2020; Zou et al., 2020; Jin et al., 2022). Therefore, caution should be exercised in predicting human clinical pharmacokinetics and pharmacodynamics solely based on rat metabolic parameters.

We found the metabolic parameters of EA and EB to have been different based on our analyses of metabolic stability and enzyme kinetics. The oxidation *t*_1/2_ of EB was shorter than that of EA, although the hydrolytic *t*_1/2_ of EB was approximately twice that of EA. Furthermore, the oxidative *V*_max_ of EA was approximately three times that of EB. Significantly more rapid hydrolysis of EA as compared to EB both in HLMs and RLMs suggest that the Z-configuration in cis-trans isomers for EA and EB was more easily hydrolyzed by carboxylesterase. The different metabolic properties of EA and EB, collectively termed stereoselectivity, were observed likely due to enzymes differing in their affinity toward chiral drugs (Lu, H., 2007; Marzo and Balant, 1996; Rentsch, 2002). As such, pharmacodynamic and/or pharmacokinetic properties of isomers require detailed evaluation in the context of clinical pharmacology (Marzo and Balant, 1996).

Identification of metabolic enzyme subtypes is essential in predicting potential drug interactions. The CYPs mediate metabolism of approximately 75% of all drugs and play a vital role in metabolic functions (Lu et al., 2015). Identification of CYP isoforms responsible for EA and EB metabolism was performed using both *in silico* methods and *in vitro* experimentation with rhCYPs. The QSAR model predicted that both isomers underwent metabolism by CYP3A4. Because different spatial configurations of isomers can lead to distinct metabolic characteristics, we then used 3D structures of EA and EB to confirm QSAR predictions *via* molecular docking. Our findings confirmed that EA and EB could directly bind CYP2C9, CYP2C19, CYP2D6 and CYP3A4, suggesting that these enzymes contain metabolic sites for these isomers. Interestingly, residues relevant to H-bond formation, H-bond number and degree of CYP isozyme binding were found to differ between EA and EB, implying that differences in metabolism among these isomers manifest on binding. Experimentation using rhCYPs further verified our prediction results. The CYP isozymes CYP1A2, CYP2C9, CYP2C19, CYP2D6 and CYP3A4 were selected because they are involved in most drug metabolism (Zhang et al., 2008). Our findings indicate that EA and EB were metabolized mainly by CYP3A4 at relative contributions of 66.20% and 58.18%, respectively, although other CYP isozymes also contributed to their metabolism to different extents.

## 5 Conclusion

Although the structures of EA and EB are similar, the metabolic characteristics of the isomers exhibit stereoselectivity. We found that rapid carboxylesterase-mediated hydrolysis of EA and EB was responsible for their rapid elimination. Importantly, significant differences were noted in metabolic parameters among humans and rats, as well as more rapid hydrolysis in rats. Finally, CYP3A4 was confirmed to be the main CYP isoform responsible for EA and EB oxidation.

## Data Availability

The raw data supporting the conclusion of this article will be made available by the authors, without undue reservation.

## Footnotes

1 FDA. M12 Drug Interaction Studies (2022). https://www.fda.gov/regulatory-information/search-fda-guidance-documents/m12-drug-interaction-studies [Accessed 26 August 2022].
